# Effects of Haplotypes of the Rice Sucrose Transporter Genes *OsSWEET11* and *OsSWEET15* on Grain Traits in Local Yunnan Germplasm Resources

**DOI:** 10.3390/ijms27125505

**Published:** 2026-06-18

**Authors:** Fahui Li, Deyu Kong, Yuxiang Li, Kun Li, Jin Xu

**Affiliations:** 1 College of Agronomy and Biotechnology, Yunnan Agricultural University, Kunming 650201, China; 2023240226@stu.ynau.edu.cn (F.L.); 2023210179@stu.ynau.edu.cn (D.K.); 2024210172@stu.ynau.edu.cn (Y.L.); 2024240242@stu.ynau.edu.cn (K.L.); 2 Rice Research Institute, Yunnan Agricultural University, Kunming 650201, China

**Keywords:** landrace rice germplasm resources, *OsSWEET15*, *OsSWEET11*, grain traits, association analysis, haplotype variation

## Abstract

The translocation of sucrose into spike grains during the grain-filling stage directly affects rice yield and quality. The sugar transporters *OsSWEET11* and *OsSWEET15* are key sucrose transporters essential for rice (*Oryza sativa* L.) grain filling. To elucidate their effects on grain traits, we analyzed sequence polymorphisms of these two genes in 139 landrace rice varieties from Yunnan, China, and conducted association and haplotype analyses. Our results indicated that grain filling degree was closely associated with grain shape, where wider grains negatively impacted grain plumpness. The association analysis revealed eight significant SNPs: six located in the coding region of *OsSWEET15* that influenced grain length, thickness, density, and 1000-grain weight (TGW), while two SNPs in *OsSWEET11* affected TGW and the thickness of milled rice grains. Haplotype analysis further validated these trait associations: *OsSWEET15* Hap2 and Hap3 conferred longer grains (with Hap2 additionally increasing TGW and Hap3 enhancing grain density/plumpness), whereas Hap1 produced narrower and thicker grains. Consistently, *OsSWEET11* Hap2 was also linked to higher TGW. The superior haplotypes identified here deepen our understanding of the genetic basis of rice grain filling and serve as potential molecular markers for marker-assisted rice breeding.

## 1. Introduction

Rice is a vital staple crop for over half of the global population [1]. The rising demand for high-quality rice has made the simultaneous improvement in yield and quality a core goal of rice breeding. Appearance quality is the initial visual impression for consumers and is a crucial commercial trait [2]. This encompasses traits such as grain length, width, thickness, the length-to-width ratio and chalkiness. These traits may also correlate with milling quality (brown rice rate, polished rice rate and whole polished rice rate), amylose content, and protein content [3]. In recent years, significant advances have been made in the research on rice grain shape, resulting in the cloning of numerous genes associated with grain shape [4]. Subsequently, further research has found that the grain filling characteristics directly determine the final grain weight of rice by regulating the accumulation of starch and protein in the endosperm, and thus affecting the formation of quality traits [5].

Grain filling involves the transportation, allocation and accumulation of photosynthetic products from source organs to sink organs [6,7,8,9]. During this process, plants utilize sucrose as the primary transported form and load it into the phloem via the apoplastic pathway. This pathway involves the transmembrane transport of sucrose and requires the assistance of sucrose transporters [10]. Consequently, proteins involved in sucrose transport play a crucial regulatory role in the grain-filling process [11]. The SWEET sugar transporter family, as a type of sugar transporter, is unique in that it does not rely on proton gradients to mediate transmembrane transport, but rather relies on sugar concentration gradients inside and outside the cell to drive it [12]. SWEET proteins can mediate bidirectional transmembrane sugar transport along the concentration gradient, driven by solute potential [12,13]. In plants, SWEET transporters mediate the efflux of sugars from source cells. Specifically, they can transport sugars from the cytosol to the apoplast (efflux) or from the apoplast into the cytosol (uptake), with the direction of transport depending on the difference in sugar concentration across the membrane.

Members of the SWEET gene family are known to be involved in the transport of sucrose. In Arabidopsis, *AtSWEET11* and *AtSWEET12* are localized to the plasma membrane, where they facilitate the efflux of sucrose from cells into the apoplast, enabling its loading into the phloem for long-distance transport [14]. Similarly, *OsSWEET11* and *OsSWEET15* also exhibit sucrose transport activity in rice. *OsSWEET11* is highly expressed in the nucellar epidermis, ovule vasculature and transverse cells during the early stages of spikelet development. The *OsSWEET11* protein plays a crucial role in regulating sucrose export from nucellar epidermal cells during grain filling, and gene-edited plants demonstrated poorly filled rice grains and reduced seed set. Furthermore, *OsSWEET11* expression has been identified in endosperm cells, indicating its involvement in transporting sucrose from the outer to the inner layers of the endosperm during the early stages of spikelet development [15]. Although both *OsSWEET15* and *OsSWEET11* regulate grain filling in rice, *OsSWEET15* exerts a much weaker individual regulatory effect on grain filling relative to *OsSWEET11*. The *OsSWEET15* mutant did not exhibit any obvious phenotypic differences compared to the wild type. However, the *OsSWEET11*/*OsSWEET15* double mutant accumulated starch in the fruit peel and was unable to develop functional endosperm within the spikelets [16]. These findings indicate that *OsSWEET11* and *OsSWEET15* play significant roles in rice grain filling [17].

Effective grain filling is crucial for enhancing both the yield and quality of rice. Identifying superior genetic resources and alleles of sugar transport genes within rice germplasm, as well as elucidating their effects on yield and quality traits, holds significant value for breeding ideal grain filling characteristics. To our knowledge, research on the impact of genes associated with grain filling on the appearance of polished rice and the rough rice grain traits remains limited.

This study utilized sequence polymorphisms of the *OsSWEET11* and *OsSWEET15* genes, as well as grain phenotypic data, from 139 landrace rice germplasm accessions in Yunnan, China. Through association analysis, we investigated the effects of these two genes on rice grain and appearance quality traits. By identifying beneficial natural variants of these genes, this study has paved the way for molecular breeding in rice.

## 2. Results

### 2.1. The Landrace Rice Germplasm in Yunnan Is Highly Diverse in Terms of Grain Traits

An analysis of the rough rice grain shape and polished rice traits in 139 landrace rice germplasm accessions from Yunnan, alongside six modern cultivated varieties, revealed significant genetic diversity within this population. Regarding rough rice grain shape, the coefficient of variation (CV) for the length-to-width ratio of the grain reached 14.35%, with a range of 1.74, indicating substantial diversity in grain shape among the accessions. This finding aligns with the relatively high CV and ranges of variation observed for both grain length and width. Furthermore, there were great differences in 1000-grain weight among the accessions, with the maximum value being more than twice that of the minimum. Additionally, significant differences were observed among the germplasm accessions in terms of rice appearance and milled rice quality traits (Table 1).

**Figure 1:**
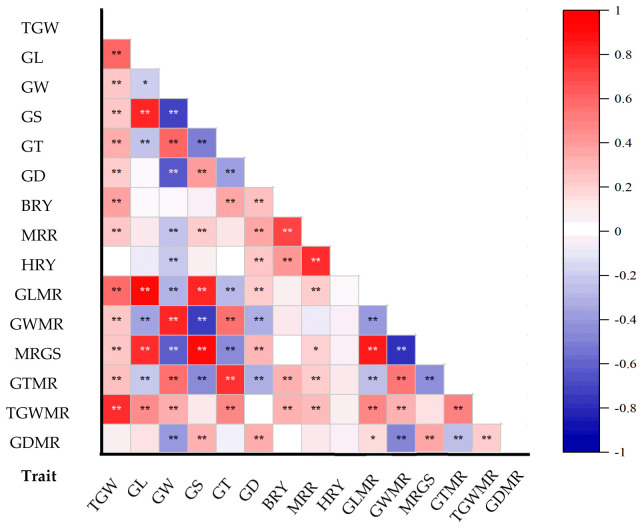
Pearson correlation analysis of rough rice traits, polished rice traits and milling quality traits. Correlation coefficients were calculated based on the mean values of three biological replicates from 145 rice accessions. Two-tailed test was used for significance analysis. “*” means *p* < 0.05 significant, “**” means *p* < 0.01 extremely significant.

Correlation analysis shows that rough rice grain shape is highly correlated with polished rice shape. While 1000-grain weight shows a highly significant positive correlation with the weight of polished rice. Furthermore, 1000-grain weight exhibits a highly significant positive correlation with grain length, grain width, grain thickness, brown rice rate, polished rice rate, and the length-to-width ratio of grains. Conversely, grain width exhibits a significant negative correlation with the polished rice ratio, brown rice ratio, grain length and grain density. Additionally, both brown and polished rice rates are extremely significantly positively correlated with grain thickness (Figure 1).

### 2.2. Sequence Variation in OsSWEET11 and OsSWEET15 Within the Germplasm Population

The *SWEET11* gene (LOC_Os08g42350) is located on chromosome 8 of rice, with a genomic length of 2854 bp. It comprises six exons and five introns, with a coding sequence (CDS) of 924 bp, encoding a protein of 307 amino acids that exhibits the typical SWEET proteins, featuring two symmetrical MtN3/saliva domains (sugar efflux transporters). The *SWEET15* gene (LOC_Os02g30910) is situated on rice chromosome 2, with a genomic length of 2746 bp. It contains six exons and five introns, with a CDS of 957 bp that encodes a plasma membrane protein of 319 amino acids.

After obtaining the gene sequences for these two genes from 139 rice germplasm accessions, SNPs with a missing rate exceeding 20% and a minor allele frequency below 0.05 were excluded. Ultimately, a total of eight SNPs were identified in *OsSWEET11* and *OsSWEET15*.

To avoid false associations, the population structure was evaluated using Structure2.2 with 80 SSR markers previously. The resulting subpopulation membership percentages were consistent with the subspecies classification [18].

All eight SNPs were found to be significantly associated with grain traits (*p* < 0.05). Specifically, two loci (SNP236 and SNP1817) were found in *OsSWEET11*, while six other loci (SNP1845, SNP1848, SNP1853, SNP1874, SNP1990 and SNP2070) were found in *OsSWEET15*. Notably, all six SNPs in *OsSWEET15* were located within the coding sequence and were strongly associated with traits such as grain length, grain thickness and 1000-grain weight. In contrast, the SNP1817 locus in *OsSWEET11* was located in the coding region and was primarily associated with 1000-grain weight. Meanwhile, the SNP236 locus in *OsSWEET11* was found in the intron region and was associated with grain thickness (Table 2).

Analysis of allelic variations in the coding sequence revealed three nonsynonymous mutations in three SNPs (SNP1853, SNP1874 and SNP1990) in *OsSWEET15*. Specifically, the base at SNP1853 changes from T to C, resulting in a change from valine to alanine. The base at SNP1874 changes from T to C, resulting in a change from isoleucine to threonine, and the base at SNP1990 changes from C to A, resulting in a change from proline to threonine (Table 3).

### 2.3. Different Haplotypes of OsSWEET15 and OsSWEET11 Are Significantly Associated with Grain Traits

#### 2.3.1. Association Analysis of *OsSWEET15* Haplotypes and Grain Traits

A haplotype-based association analysis of grain traits revealed significant differences in terms of grain length, length-to-width ratio, grain thickness and 1000-grain weight among the *OsSWEET15* germplasm resources.

**Figure 2:**
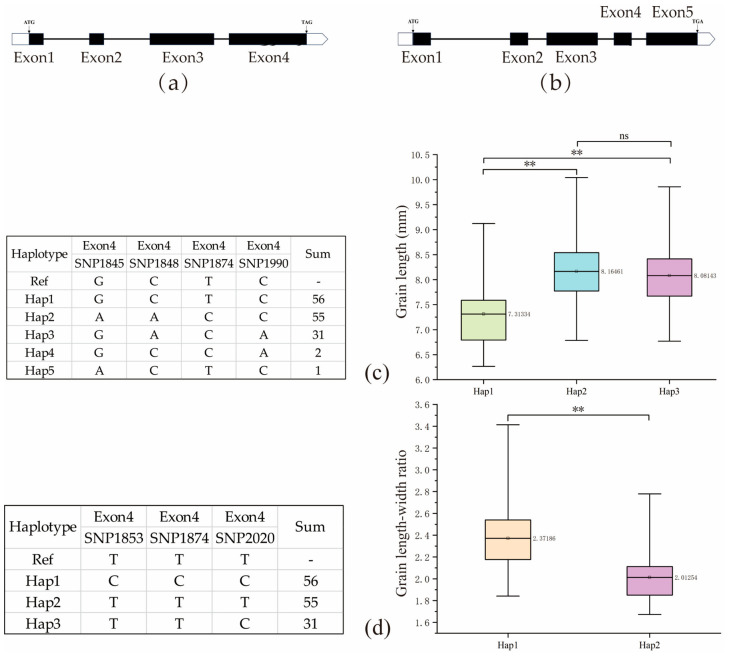

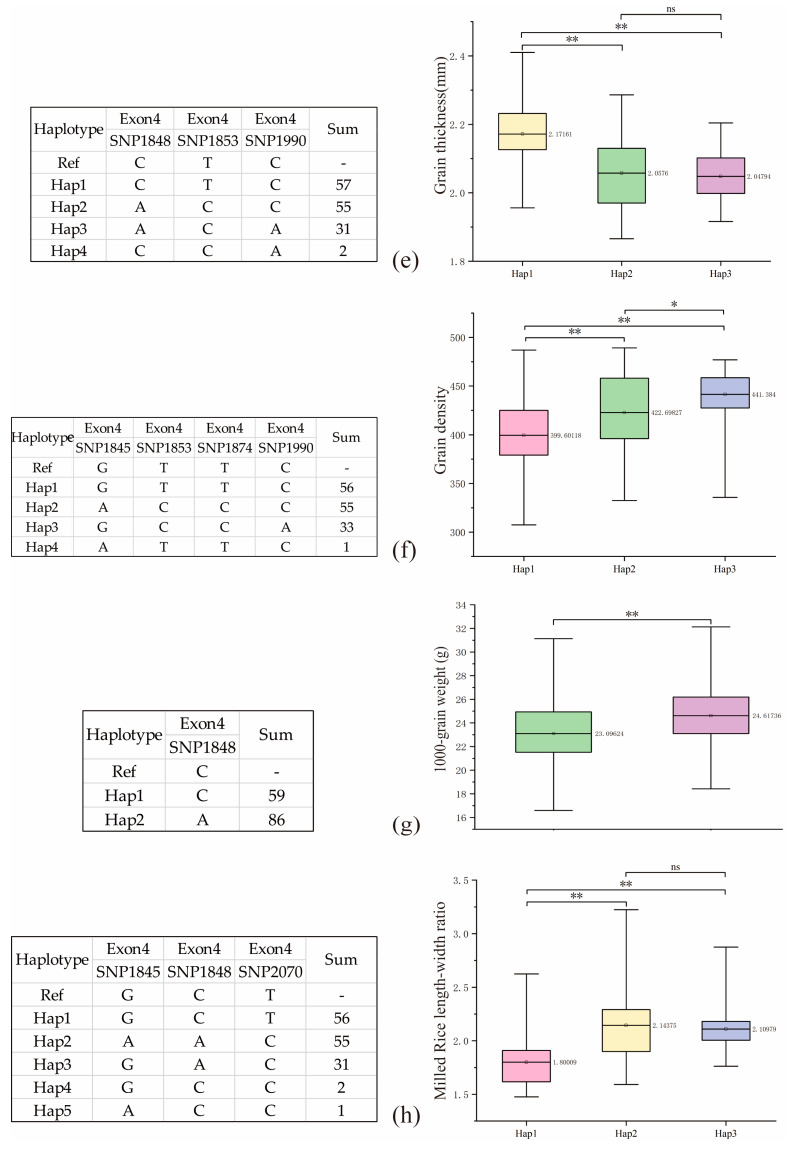

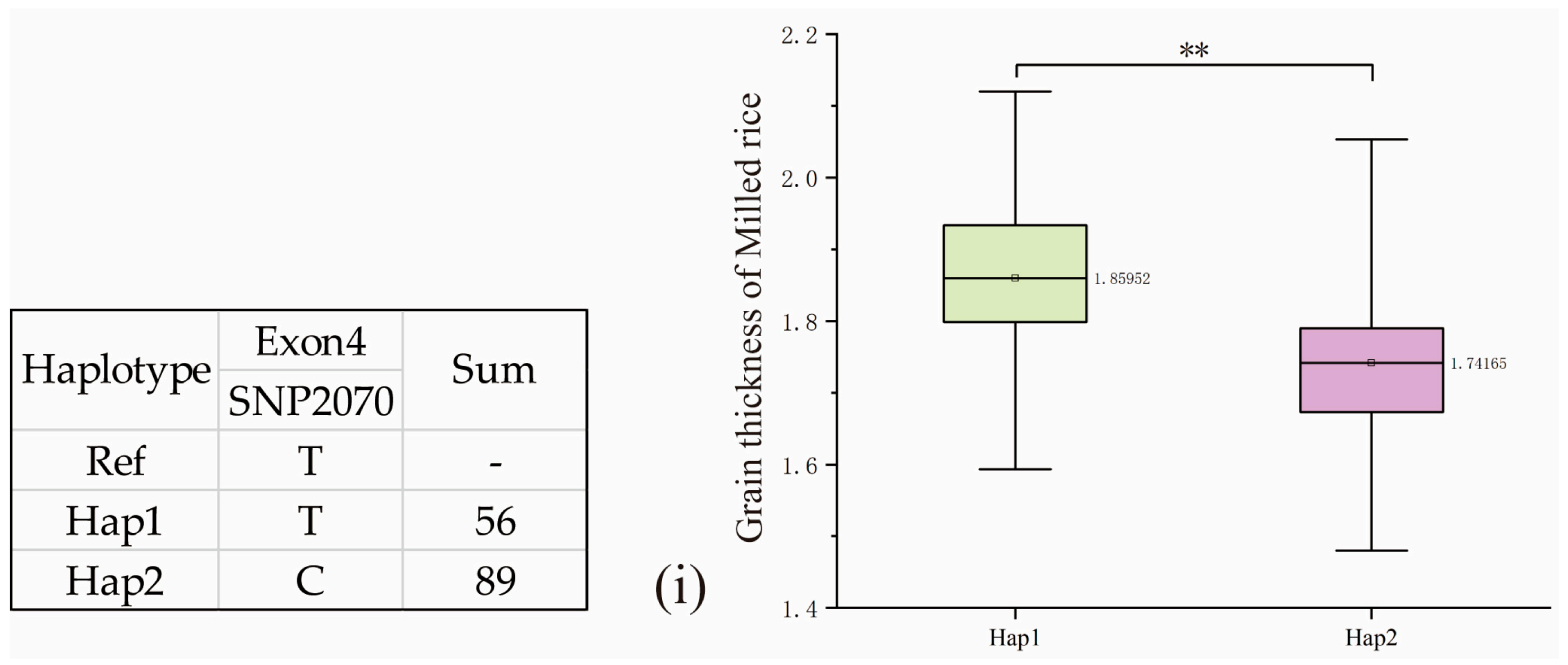
The associations between the *OsSWEET15* haplotypes and grain traits. (**a**) For the *OsSWEET15* gene structure diagram; (**b**) *OsSWEET11* gene structure diagram; (**c**) haplotype analysis of *OsSWEET15* and its association with grain length; (**d**) haplotype analysis of *OsSWEET15* and its association with grain length-to-width ratio; (**e**) haplotype analysis of *OsSWEET15* and its association with grain thickness; (**f**) haplotype analysis of *OsSWEET15* and its association with grain density; (**g**) haplotype analysis of *OsSWEET15* and its association with 1000-grain weight; (**h**) haplotype analysis of *OsSWEET15* and its association with Milled rice length-to-width ratio; (**i**) haplotype analysis of *OsSWEET15* and its association with Milled rice thickness. The left side of each figure shows the gene structure of *OsSWEET15* and the polymorphic SNP loci that constitute the haplotype; the “Sum” column indicates the number of germplasm accessions carrying this haplotype within the population. The box-and-whisker plot on the right illustrates the association analysis between haplotype and grain phenotypes; the values are presented as mean ± standard deviation (SD) from the same haplotype landraces. Differences between multiple haplotypes were analyzed by one-way analysis of variance (ANOVA) followed by Duncan’s multiple range test. *: Significant differences (*p* < 0.05); **: highly significant (*p* < 0.01); ns: no significant differences.

Five *OsSWEET15* haplotypes were associated with grain length. Accessions with haplotypes Hap2 and Hap3 were found to have longer grains, exhibiting significant differences (*p* < 0.01) in grain length compared to accessions carrying the Hap1 haplotype. In contrast, only two and one accessions comprised haplotypes Hap4 and Hap5, respectively (Figure 2a).

Three haplotypes were associated with the grain length-to-width ratio, where the ratio of accessions with haplotype Hap1 was significantly greater than that of accessions with haplotype Hap2 (Figure 2b).

Among the four haplotypes associated with grain thickness, accessions with Hap1 exhibited significantly thicker grains (Figure 2c).

Among the four haplotypes associated with grain density, significant differences in grain density were observed between Hap1, Hap2 and Hap3. In particular, the germplasm carrying Hap3 shows higher grain density, suggesting that this germplasm may possess superior grain-filling characteristics (Figure 2d).

Of the two haplotypes associated with 1000-grain weight, accessions carrying Hap2 exhibited significantly higher values than those carrying Hap1.

Regarding polished rice grain shape, *OsSWEET15* exhibits five haplotypes that differ significantly in polished rice length-to-width ratio, with relatively few accessions carrying Hap4 and Hap5. Accessions carrying Hap2 and Hap3 had an extremely significantly greater polished rice length-to-width ratio than those carrying Hap1 (Figure 2f).

Among the two haplotypes associated with polished rice thickness, the thickness of Hap1 was significantly greater than that of the Hap2 haplotype (Figure 2g).

#### 2.3.2. Association Analysis of *OsSWEET11* Haplotypes and Grain Traits

**Figure 3:**
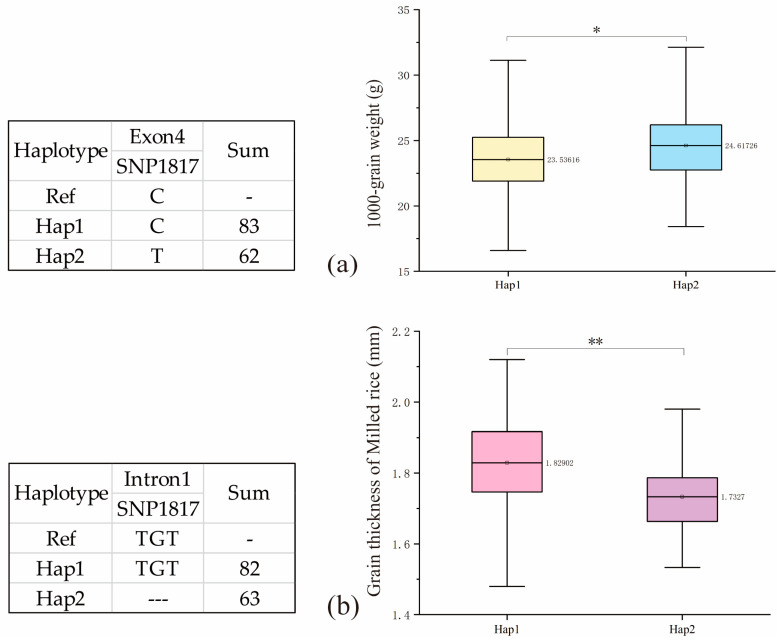
The associations between the *OsSWEET11* haplotypes and grain traits. (**a**) *OsSWEET11* haplotype analysis and association analysis with 1000-grain weight; (**b**) *OsSWEET11* haplotype analysis and association analysis with Milled rice thickness. The left side of each figure shows the gene structure of *OsSWEET11* and the polymorphic SNP loci that constitute the haplotype; the “Sum” column indicates the number of germplasm accessions carrying this haplotype within the population. The box-and-whisker plot on the right illustrates the association analysis between haplotype and grain phenotypes; the values are presented as mean ± standard deviation (SD) from the same haplotype landraces. Differences between multiple haplotypes were analyzed by one-way analysis of variance (ANOVA) followed by Duncan’s multiple range test. *: Significant differences (*p* < 0.05); **: highly significant (*p* < 0.01); ns: no significant differences.

Analysis of haplotypes for the *OsSWEET11* gene revealed that two haplotypes are associated with 1000-grain weight. Accessions of haplotype Hap2 exhibited significantly higher 1000-grain weights compared to those of haplotype Hap1 (Figure 3a).

Furthermore, among the two haplotypes associated with grain thickness, haplotype Hap1 demonstrated a highly significant increase in grain thickness compared to haplotype Hap2 (Figure 3b).

## 3. Discussion

Our study demonstrates that the landrace rice germplasm in Yunnan exhibits substantial genetic diversity in terms of grain traits, rice appearance, and milling quality characteristics. Furthermore, grain filling is closely associated with grain shape [19]. Correlation analysis revealed an extremely significant positive correlation between the 1000-grain weight and the grain length-to-width ratio. This indicates that within the landrace rice germplasm resources, slender grains (characterized by larger length-to-width ratios) tend to have a higher grain weight, suggesting denser filling [20]. This finding is consistent with the observation of a highly significant negative correlation between grain width and the brown rice rate, polished rice rate and grain density. This suggests that wider grains are less favorable for optimal rice grain filling.

*OsSWEET11* and *OsSWEET15* are both sucrose transporters associated with grain filling. Notably, mutations in *OsSWEET11* can lead to significant hindrances in grain filling, while mutations in *OsSWEET15* exhibit no apparent phenotypic alterations [21]. However, the double mutant of *OsSWEET11* and *OsSWEET15* demonstrates severe defects in grain filling, suggesting that *OsSWEET11* serves as the primary protein for sucrose transport during this process, exerting a substantial influence on grain sugar transport. Conversely, *OsSWEET15* appears to be a minor-effect gene that collaborates with *OsSWEET11* in rice grain filling.

Our findings further support this result. Among the variation sites identified in *OsSWEET11*, only one was located in the coding region, and it was a synonymous mutation. This observation aligns with the key role of *OsSWEET11* in regulating sucrose transport during grain filling; it has likely undergone significant selective pressure, as mutations in the coding region would impair the functionality of sucrose transport, resulting in the retention of the gene sequence through evolution. In contrast, six variation sites were identified in the *OsSWEET15* coding sequence, three of which were non-synonymous mutations. This suggests that *OsSWEET15*, functioning as a minor-effect sucrose transporter during grain filling, has experienced weaker purifying selection than *OsSWEET11* throughout evolution, allowing it to accumulate greater genetic variation and thereby enrich the genetic diversity of rice populations. Importantly, not all of this variation is neutral: we found that polymorphisms at these loci were significantly associated with changes in grain length, length-to-width ratio, grain thickness and grain density. Among these functional variations, three non-synonymous mutations in the coding region are particularly noteworthy: Ala123Val (SNP1234, G → T), Ile187Thr (SNP1874, T → C), and Pro245Thr (SNP1990, C → A). All three mutations are located in the central sucrose-binding pocket of *OsSWEET15*, but exert distinct effects on protein function through different molecular mechanisms: the Ala-Val mutation reduces the volume of the binding pocket and increases substrate binding specificity; the Ile-Thr mutation introduces an additional hydrogen bond with sucrose, significantly enhancing substrate binding affinity and leading to increased total sugar accumulation in grains; and the Pro-Thr mutation increases the conformational flexibility of the binding pocket exit, accelerating sucrose release into the apoplast and improving grain filling uniformity. These distinct functional effects directly explain the divergent phenotypic performances of *OsSWEET15* haplotypes: Hap2 carrying the Ile187Thr mutation shows significantly higher 1000-grain weight, while Hap3 carrying the Pro245Thr mutation exhibits markedly higher grain density.

This pattern indicates that *OsSWEET15* has accumulated diverse functional genetic variations through both natural and artificial selection, which enables rice to adapt to different grain-filling patterns and grain type requirements. However, the minor effects of individual *OsSWEET15* variants result in subtle phenotypic changes that are often subtle and easily masked by environmental factors or the influence of major genes. This makes traditional genetic methods, such as map-based cloning, single-gene mutation and gene editing, inadequate for accurately dissecting its function. Consequently, candidate gene association analysis, which can detect small-effect genetic variations in natural populations, emerges as a particularly powerful tool for elucidating the function of minor-effect genes like *OsSWEET15*. A critical consideration in all candidate gene association studies is the potential impact of population stratification, which can lead to spurious associations between genetic markers and traits. To address this issue, we used the mixed linear model (MLM) that simultaneously incorporates both the population structure matrix (Q matrix) derived from STRUCTURE analysis and the pairwise kinship matrix (K matrix) for all association tests.

Germplasm resources harboring superior haplotypes are crucial genetic resources for crop breeding [22,23]. Association analysis can be used to explore the relationships between target genes and associated traits. When integrated with haplotype analysis, association analysis enables the identification of natural variant sites and advantageous haplotypes that contribute positively to the phenotype. Our study demonstrated that the 1000-grain weight of the Hap2 haplotype of *OsSWEET11* is significantly higher than that of other haplotypes; however, whether this increase is related to the haplotype’s capacity to enhance grain filling requires further investigation. Furthermore, the Hap2 haplotype of *OsSWEET15* was found to result in a higher 1000-grain weight. Germplasm possessing the Hap3 haplotype showed significant advantages in terms of both grain and polished rice density, suggesting that germplasm carrying this haplotype experiences more effective grain filling. Furthermore, germplasm containing both Hap2 and Hap3 had a larger length-to-width ratio, resulting in slimmer grains and improved appearance quality.

Our study elucidated the effects of natural variation in *OsSWEET11* and *OsSWEET15* on the grain-related characteristics of landrace rice germplasm resources in Yunnan, identifying superior haplotypes and their breeding value.

Although the role of *OsSWEET11* and *OsSWEET15* in regulating rice grain traits has been effectively validated, this study has several limitations that should be acknowledged: This study relied solely on natural population association and haplotype analysis, without conducting gene expression profiling analysis, subcellular localization analysis, or transgenic/CRISPR knockout/overexpression experiments to validate the effects of identified SNPs and haplotypes on *OsSWEET15*/*OsSWEET11* expression, protein function, and sucrose transport activity. Therefore, there is a lack of direct molecular evidence linking sequence variations to biological function; all phenotype data were collected from plants grown in greenhouses in Kunming, and grain traits are highly sensitive to environmental factors such as temperature, light, and soil fertility [24,25]. Therefore, the lack of multi-environment field experiments limits the generalizability of our research results, and the genetic effects of SNP/haplotypes identified under field conditions are still unclear. The natural population used here possesses a fixed sample size, and the restricted germplasm resource may overlook rare functional allelic variants with minor genetic effects. Moreover, the current analysis mainly focuses on the individual genetic effect of *OsSWEET11*/*OsSWEET15*, and epistatic interactions between these two SWEET genes or other grain-filling-related loci were not systematically explored, which restricts a comprehensive interpretation of the genetic regulatory network underlying rice grain development.

Germplasm resources carrying superior haplotypes are crucial genetic resources for crop breeding [26]. Our study identified *OsSWEET15* Hap2 (high 1000-grain weight) and Hap3 (high grain density) as elite haplotypes for rice grain quality and yield improvement, as well as *OsSWEET11* Hap2 for higher grain weight. These favorable haplotypes provide valuable molecular markers for marker-assisted selection (MAS) in rice breeding. These markers can be applied to early-generation seedling screening in rice breeding practice, effectively avoiding the blindness of traditional phenotypic selection, shortening the breeding cycle, and improving the accuracy of targeted selection for high-yield and high-quality rice varieties.

Overall, this study systematically described the natural genetic variations of *OsSWEET11* and *OsSWEET15* in different rice germplasm populations, and successfully identified multiple elite haplotypes closely related to grain weight and density traits. This work provides new haplotype resources and candidate functional SNPs, further enriching the genetic basis of rice grain filling and yield variation, providing valuable germplasm resources and molecular markers for precise improvement in rice yield and quality, and providing clear and feasible goals for subsequent gene function research and molecular breeding.

## 4. Materials and Methods

### 4.1. Materials

The rice accessions used in the study included 139 landraces from Yunnan, China, as well as six modern cultivated varieties. Of these, 89 were japonica, and 56 were indica (Appendix A.1).

### 4.2. Measurement of Grain Traits

All rice accessions were cultivated in a greenhouse in Kunming, Yunnan (102°44′ E, 25°7′ N). Once mature, each variety was harvested. The rice grains were sun-dried and stored in a room for three months until their physical and chemical characteristics had stabilized. The grain traits assessed included grain length, width, thickness, the length-to-width ratio and the 1000-grain weight. Appearance quality traits measured included polished rice length, width, length-to-width ratio, chalky grain ratio, brown rice ratio, polished rice ratio, and 1000-grain weight of polished rice, according to the standard NY/T83-2017 issued by the Chinese Ministry of Agriculture for measurement and analysis. Measurement of brown rice yield is as follows: weigh 50.00 g of dried rice grains (with a moisture content controlled at approximately 12%) (error ≤ 0.02), use a huller (Kett Electric Laboratory Co. Ltd., Tokyo, Japan) to remove the shell, and then weigh the weight of the resulting brown rice; for precision rice rate, weigh approximately 20 g of ground brown rice, use a precision rice mill (Shanghai Qingpu Lüzhou Instrument Co., Ltd., Shanghai, China) to grind it, use a 100-mesh sieve to remove surface dust, weigh it and calculate the precision rice rate; for whole rice rate, weigh approximately 10 g of the obtained polished rice, select incomplete grains (grain length ≤ 3/4), miscellaneous grains, and yellow grains from the polished rice, weigh them, and calculate the whole rice rate.

The measurement of appearance quality involves taking 10 healthy, plump, and uniformly sized intact grains from the grain/milled rice, arranging them end-to-end above a ruler and measuring them with a Vernier caliper (Harbin Measuring & Cutting Tool Group Co., Ltd., Harbin, China), with an accuracy of 0.01 mm and a repetition error of ≤0.50 mm. Each material is repeated three times, and the average of the three measurements is taken as the final value.

### 4.3. Gene Sequence Analysis

#### 4.3.1. Genomic DNA Extraction

Collect fresh young leaves from 2-week-old rice seedlings of each variety, immediately freeze them in liquid nitrogen, and store them at −80 °C until use. Genomic DNA was extracted using an improved cetyltrimethylammonium bromide (CTAB) method.

#### 4.3.2. PCR Amplification of Target Fragment

Based on the Rice Annotation Project Database, primers were designed based on the sequences of *OsSWEET11* (LOC_Os08g42350, 2854 bp) and *OsSWEET15* (LOC_Os02g30910, 2746 bp) (Table 4). PCR amplification was carried out in a 25 μL reaction system, which included: 12.5 μL 2× Taq PCR Master Mix (from BioNTech (Shanghai) Co., Ltd., Shanghai, China), 1 μL forward primer (10 μM), 1 µL reverse primer (10 µM) (synthesized by BioNTech (Shanghai) Co., Ltd.), 1 μL genomic DNA template, and 9.5 μL nuclease-free water.

The PCR reaction procedure is as follows: initial denaturation at 94 °C for 5 min; denaturate for 30 s and 35 cycles at 94 °C, annealing time for 30 s, annealing temperature 58 °C, extend for 1 min/kb at 72 °C, and finally extend for 10 min at 72 °C. PCR products were detected by electrophoresis on 1.5% agarose gel stained with ethidium bromide (EB) and observed under an gel documentation system (Shanghai Tanon Science & Technology Co., Ltd., Shanghai, China) to confirm whether there were specific amplicons of the expected size. After PCR amplification, qualified PCR products were submitted to Biotechnology (Shanghai) Co., Ltd. (Shanghai, China) for Sanger sequencing using an Applied Biosystems™ 3730XL DNA Analyzer (Thermo Fisher Scientific, Waltham, MA, USA). Raw sequencing chromatograms were assembled and trimmed via SnapGene; multiple sequence alignment of *OsSWEET11* and *OsSWEET15* was performed with MEGA11 to screen valid SNP variants after removing low-quality and missing-data sites.

### 4.4. Statistical Analysis

The phenotypic data were organized using Microsoft Excel 2019 (Microsoft Corporation, Redmond, WA, USA). SnapGene (version 6.2.1, GSL Biotech LLC, Chicago, IL, USA) was used to assemble the sequencing reads, and MEGA11(version 11.0.13, Institute for Genomics and Evolutionary Medicine, Temple University, Philadelphia, PA, USA) was used for sequence alignment. We performed significance tests on the data differences to analyze genetic sequence polymorphisms using SPSS 27.0 (IBM Corp., Armonk, NY, USA). Haplotype analysis of significantly associated polymorphic loci was performed using DnaSP6 (version 6.12.03, Universitat de Barcelona, Barcelona, Spain) and Excel to investigate the association between genotypic haplotypes and phenotypic traits. Within the specified analysis interval, all samples with identical nucleotide sequences are classified as the same haplotype (Haplotype, Hap), and there is at least one nucleotide difference (SNP or Indel) between different haplotypes. In addition, OriginPro 2024 (64-bit) SR1 version 10.1.0.178 (OriginLab Corporation, Northampton, MA, USA) was used for data visualization. The mixed linear model (MLM) program in the TASSEL5 software (version 5.2.93, Cornell University, Ithaca, NY, USA) was used to perform correlation analysis between selected genes and measured phenotypic traits. The associated region is the coding region, and the most polymorphic and relatively concentrated fragments are selected. The Q matrix is obtained by running the Structure [27] software (version 2.3.4, Stanford University, Stanford, CA, USA), and the K matrix is calculated by SPAGeDi software (version 1.5, Université Libre de Bruxelles, Brussels, Belgium). A population structure analysis on the obtained Q matrix and K matrix was conducted, and it was used as a covariate to correct the data and reduce the influence of kinship on the results [28,29].

## 5. Conclusions

The landrace rice germplasm resources in Yunnan exhibited rich genetic diversity in terms of grain traits. Correlation analysis of these traits revealed that grain filling was closely related to grain shape. This indicates that varieties with wider grains are less favorable for achieving full grain filling in rice.

A total of eight significantly associated SNP loci were identified in the association analysis. Of these, six coding-region loci were found in *OsSWEET15*, which exerted significant effects on traits such as grain length, grain thickness, grain density and 1000-grain weight. *OsSWEET11* harbored two loci, one of which was situated in the coding region.

Different haplotypes of *OsSWEET15* exhibited significant associations with rough rice weight and shape traits: germplasms carrying the Hap1 haplotype had a higher grain length-to-width ratio and produced slender rough grains, those with the Hap3 haplotype showed higher grain density and better grain filling characteristics, and those harboring the Hap2 haplotype exhibited higher TGW. Similarly, germplasms carrying the Hap2 haplotype of *OsSWEET11* also tended to have higher TGW. These results suggest that natural variations in *OsSWEET11* and *OsSWEET15* may be involved in regulating rice grain trait formation, and the identified superior haplotypes provide potential molecular markers for rice breeding.

## Figures and Tables

**Table 1:** Variation in grain-related traits of landrace rice germplasm in Yunnan.

Trait ^a^	MIN	MAX	AV	SD	CV(%)
TGW (g)	16.60	32.13	24.00	2.81	11.71
GL (mm)	6.27	10.04	7.80	0.77	9.87
GW (mm)	2.63	4.23	3.52	0.28	7.95
GS	1.67	3.41	2.23	0.32	14.35
GT (mm)	1.87	2.41	2.10	0.11	5.24
GD (g/L)	307.42	489.24	417.95	38.10	9.12
BRY (%)	68.23	85.41	78.36	2.98	3.80
MRR (%)	51.13	76.78	67.08	4.02	5.99
HRY (%)	39.96	76.58	63.16	5.70	9.02
GLMR (mm)	4.28	7.42	5.45	0.56	10.28
GWMR (mm)	2.02	3.23	2.75	0.22	8.00
MRGS	1.48	3.22	2.00	0.33	16.50
GTMR (mm)	1.48	2.12	1.79	0.12	6.70
TGWMR (g)	11.95	25.20	18.44	2.36	12.80
GDMR (g/L)	479.84	787.38	691.86	49.11	7.10

All traits were measured with three biological replicates. Values are presented as minimum (MIN), maximum (MAX), mean (AV), standard deviation (SD) and coefficient of variation (CV, %) of each trait. ^a^ TGW, 1000-grain weight. GL, grain length. GW, grain width. GS, grain shape (length-to-width ratio). GT, grain thickness. GD, grain density. BRY, brown rice yield. MRR, milled rice recovery. HRY, head rice yield. GLMR, grain length of milled rice. GWMR, grain width of milled rice. MRGS, milled rice grain shape. GTMR, grain thickness of milled rice. TGWMR, 1000-grain weight of milled rice. GDMR, grain density of milled rice. The same below.

**Table 2:** SNPs associated with grain traits.

Gene	Trait	SNP Site	*p*-Value	Gene	Trait	SNP Site	*p*-Value
*OsSWEET15*	GL	1845	1.00 × 10^−20^	*OsSWEET15*	MRGS	1874	0.022448
*OsSWEET15*		1848	2.54 × 10^−39^	*OsSWEET15*		2070	0.014485
*OsSWEET15*		1874	2.00 × 10^−30^	*OsSWEET15*	GD	1845	0.006925
*OsSWEET15*		1990	7.21 × 10^−8^	*OsSWEET15*		1853	2.26 × 10^−12^
*OsSWEET15*	GT	1848	0.003198	*OsSWEET15*		1874	4.39 × 10^−5^
*OsSWEET15*		1853	6.83 × 10^−21^	*OsSWEET15*		1990	6.76 × 10^−5^
*OsSWEET15*		1990	2.45 × 10^−12^				
*OsSWEET15*	TGW	1848	2.85 × 10^−5^	*OsSWEET11*	TGW	1817	0.047214
*OsSWEET15*	GS	1853	8.22 × 10^−4^	*OsSWEET11*	GTMR	236	0.01243

**Table 3:** Allelic variants in the coding sequence of *OsSWEET11* and *OsSWEET15*.

Gene	Position in the CDS Region/bp	Base Variation ^a^	Codon Variation
*OsSWEET11*	SNP1817	C/T	UAC/UAU
*OsSWEET15*	SNP1845	G/A	UCG/UCA
	SNP1848	C/A	UCC/UCA
	SNP2070	T/C	GCU/GCC
	SNP1853	T/C	GUG/GCG
	SNP1874	T/C	AUC/ACC
	SNP1990	C/A	CCC/ACC

^a^ A, T, C, G, represent adenine, thymine, guanine, cytosine, respectively.

**Table 4:** Sequence of primers used in the study.

Primer Name	Primer Sequence	Annealing Temperature (℃)
*OsSWEET11*-1F	CTGGCTAGTTTCTAGCTGGTGTC	58
*OsSWEET11*-1R	TTGTACACCTGCAAGAACGTCG	
*OsSWEET11*-2F	CATCTCCTTCCTGGTGTTCCTTG	58
*OsSWEET11*-2R	TCTGTCGTCGTCGAGATCAGTC	
*OsSWEET15*-F	CTCAAGAAGGACGTGTTCGTGG	58
*OsSWEET15*-R	GACTACACTTCCACAACAATGGCC	

## Data Availability

The data supporting the findings of this study are available within the article.

## Abbreviations

The following abbreviations are used in this manuscript:

TGW	1000-grain weight
GW	Grain width
GL	Grain length
GS	Grain shape (length-to-width ratio)
GT	Grain thickness
GD	Grain density
BRY	Brown rice yield
MRR	Milled rice recovery
HRY	Head rice yield
GLMR	Grain length of Milled rice
GWMR	Grain width of Milled rice
MRGS	Milled Rice Grain shape
GTMR	Grain thickness of Milled rice
TGWMR	1000-grain weight of Milled rice
GDMR	Grain density of Milled rice
SNP	Single nucleotide polymorphism
bp	Base pair
DNA	Deoxyribonucleic Acid
CTAB	Cetyltrimethylammonium bromide
V/A/I	Valine/Alanine/Isoleucine
T/P/S	Threonine/Proline/Serine
C/Y	Cysteine/Tyrosine

## Appendix A

### Appendix A.1

**Table A1:** Yunnan rice landraces resources.

Serial Number	ID	Variety	Origin	Subpopulation
1	LR-1	Yu Nuo	Yunnan	Indica rice
2	LR-2	Hongpi Nuo	Yunnan	Japonica rice
3	LR-3	Sanbang Bang	Yunnan	Indica rice
4	LR-4	Zhongguo Hongbiangu	Yunnan	Japonica rice
5	LR-6	Guangyedao 1	Yunnan	Japonica rice
6	LR-7	Zao Nuo 1 Hao	Yunnan	Japonica rice
7	LR-8	Hongqiao Huigu	Yunnan	Japonica rice
8	LR-9	Maxian Gu	Yunnan	Japonica rice
9	LR-10	Guanshan Gu	Yunnan	Indica rice
10	LR-11	Hongmaoying	Yunnan	Japonica rice
11	LR-12	Hongmaoying	Yunnan	Japonica rice
12	LR-13	Youzhi Changli Jing	Yunnan	Japonica rice
13	LR-14	Huangke Nuo	Yunnan	Indica rice
14	LR-15	Huangke Nuo	Yunnan	Indica rice
15	LR-16	Tengchong Lufenggu	Yunnan	Indica rice
16	LR-17	Ebuqin	Yunnan	Japonica rice
17	LR-18	Xiaoma Gu	Yunnan	Japonica rice
18	LR-19	Shuiza Gu	Yunnan	Indica rice
19	LR-20	Qiannani	Yunnan	Indica rice
20	LR-22	Liuyue Gu	Yunnan	Indica rice
21	LR-23	Liandao Gu	Yunnan	Japonica rice
22	LR-25	Shala Gu	Yunnan	Indica rice
23	LR-26	Xi Gu	Yunnan	Indica rice
24	LR-27	Lübang Gu	Yunnan	Indica rice
25	LR-28	Laoshuya	Yunnan	Indica rice
26	LR-29	P1-238-2	Yunnan	Indica rice
27	LR-30	P1-522	Yunnan	Japonica rice
28	LR-31	Hua’er Han	Yunnan	Indica rice
29	LR-32	Yongshan Hangu	Yunnan	Indica rice
30	LR-33	Liuyue Gu	Yunnan	Indica rice
31	LR-34	LR-34	Yunnan	Japonica rice
32	LR-35	Aijiao Nuo	Yunnan	Indica rice
33	LR-36	Mengbai Xiaohonggu	Yunnan	Japonica rice
34	LR-37	Paozhu Gu	Yunnan	Indica rice
35	LR-38	Hongjiao Gu	Yunnan	Indica rice
36	LR-39	Dawan Gu	Yunnan	Japonica rice
37	LR-40	Changmanggu	Yunnan	Japonica rice
38	LR-41	Hongangu	Yunnan	Japonica rice
39	LR-42	Guangyedao 2	Yunnan	Japonica rice
40	LR-43	Liandaogu	Yunnan	Japonica rice
41	LR-44	Yunlu 52	Yunnan	Japonica rice
42	LR-45	Honganlao	Yunnan	Japonica rice
43	LR-46	Chenganxiang	Yunnan	Japonica rice
44	LR-48	Bainaqian	Yunnan	Indica rice
45	LR-49	Banmanggu	Yunnan	Japonica rice
46	LR-50	Yunlu 54	Yunnan	Japonica rice
47	LR-51	Maqiangu	Yunnan	Indica rice
48	LR-53	Ebendian	Yunnan	Japonica rice
49	LR-54	Maqian	Yunnan	Indica rice
50	LR-55	Qianni Hongmi	Yunnan	Indica rice
51	LR-56	Wuyu 28	Yunnan	Japonica rice
52	LR-57	Zhaotong Xiaomagu	Yunnan	Japonica rice
53	LR-58	Mengwanggu	Yunnan	Japonica rice
54	LR-59	Yongning Xiaobaigu	Yunnan	Japonica rice
55	LR-60	Wuzui Honggu	Yunnan	Indica rice
56	LR-61	Hainangu	Yunnan	Indica rice
57	LR-62	Mazhagu	Yunnan	Indica rice
58	LR-63	Dibaigu-2	Yunnan	Japonica rice
59	LR-64	Baihaigu	Yunnan	Japonica rice
60	LR-65	Daxianggu-2	Yunnan	Japonica rice
61	LR-66	Guangyedao 3	Yunnan	Japonica rice
62	LR-67	Dabaigu	Yunnan	Indica rice
63	LR-68	Daxianggu-3	Yunnan	Japonica rice
64	LR-69	Yongning Qunxuan 2 Hao	Yunnan	Japonica rice
65	LR-70	Zhushinuo	Yunnan	Japonica rice
66	LR-71	Bendi Hangu	Yunnan	Japonica rice
67	LR-72	Lufeng Nugu	Yunnan	Japonica rice
68	LR-73	Lufeng Nugu	Yunnan	Indica rice
69	LR-74	Qiantuohe	Yunnan	Indica rice
70	LR-75	Yongning Xiaomagu	Yunnan	Japonica rice
71	LR-76	Yunkang 09	Yunnan	Japonica rice
72	LR-77	Dalaka Honggu	Yunnan	Japonica rice
73	LR-78	Dengleng	Yunnan	Indica rice
74	LR-79	LR-79	Yunnan	Indica rice
75	LR-80	Fuha Hani	Yunnan	Japonica rice
76	LR-81	Hongjiao Xiangjing	Yunnan	Indica rice
77	LR-82	P1-229	Yunnan	Indica rice
78	LR-83	Maqian	Yunnan	Indica rice
79	LR-84	Lengshui Gu	Yunnan	Indica rice
80	LR-85	Laolai Huang	Yunnan	Indica rice
81	LR-86	Shuangbai Gu	Yunnan	Indica rice
82	LR-87	Xianwei Dao	Yunnan	Indica rice
83	LR-88	Xiang Gu	Yunnan	Japonica rice
84	LR-89	Heilengshui	Yunnan	Japonica rice
85	LR-90	Langcang Dabaigu	Yunnan	Japonica rice
86	LR-91	Langcang Dabaigu	Yunnan	Indica rice
87	LR-92	Langcang Dabaigu	Yunnan	Japonica rice
88	LR-93	Hua Gu	Yunnan	Japonica rice
89	LR-94	Wanli Xiang	Yunnan	Japonica rice
90	LR-95	Xiaohuang Nuo	Yunnan	Japonica rice
91	LR-96	Maqian Gu	Yunnan	Indica rice
92	LR-97	Luoge Maqian	Yunnan	Japonica rice
93	LR-98	Daxianggu-1	Yunnan	Japonica rice
94	LR-99	Achuche (Lengshui Gu)	Yunnan	Indica rice
95	LR-100	Laolai Hong 1	Yunnan	Indica rice
96	LR-102	Xiao Aijiao	Yunnan	Japonica rice
97	LR-103	Maqing	Yunnan	Japonica rice
98	LR-104	Maqing	Yunnan	Indica rice
99	LR-105	Huangsi Nuo Gu	Yunnan	Japonica rice
100	LR-106	Tian 1087	Yunnan	Indica rice
101	LR-107	Han Gu	Yunnan	Indica rice
102	LR-109	Manchecheran	Yunnan	Japonica rice
103	LR-110	Wadu Xiaobaigu	Yunnan	Japonica rice
104	LR-111	Pannong 1 Hao	Yunnan	Indica rice
105	LR-112	LR-114	Yunnan	Japonica rice
106	LR-113	Xiao Honggu	Yunnan	Japonica rice
107	LR-114	YL1311	Yunnan	Japonica rice
108	LR-115	M1-448 (Yongning Laopinzhong)	Yunnan	Japonica rice
109	LR-116	M1-429 (Yongning Laopinzhong)	Yunnan	Indica rice
110	LR-117	Xiaobaigu 1	Yunnan	Japonica rice
111	LR-118	Xiaohonggu	Yunnan	Japonica rice
112	LR-119	Huaaigu 2	Yunnan	Japonica rice
113	LR-120	Xiaobaigu 2	Yunnan	Japonica rice
114	LR-122	Ersigu	Yunnan	Indica rice
115	LR-123	Lijiang Xintuan Heigu	Yunnan	Indica rice
116	LR-124	Yongning Dabaigu	Yunnan	Japonica rice
117	LR-125	Yongning Wadu Xiaobaigu	Yunnan	Indica rice
118	LR-126	Misha Honggu	Yunnan	Japonica rice
119	LR-127	Long 16	Heilongjiang	Japonica rice
120	LR-128	Long 16-2	Heilongjiang	Japonica rice
121	LR-129	LR129	Yunnan	Japonica rice
122	LR-130	LR-18	Yunnan	Japonica rice
123	LR-131	LR139	Yunnan	Japonica rice
124	LR-132	LR140	Yunnan	Japonica rice
125	LR-133	LR141	Yunnan	Japonica rice
126	LR-134	LR142	Yunnan	Japonica rice
127	LR-135	LR143	Yunnan	Japonica rice
128	LR-136	Jinning Xianggu	Yunnan	Japonica rice
129	LR-137	Hongmi	Yunnan	Japonica rice
130	LR-138	Chenu	Yunnan	Japonica rice
131	LR-139	US19	USA	Japonica rice
132	LR-140	US30	USA	Japonica rice
133	LR-141	Menghai Xianggu	Yunnan	Japonica rice
134	LR-142	Lanangu	Yunnan	Japonica rice
135	LR-143	Daxiangnuo	Yunnan	Japonica rice
136	LR-144	Kaogougu	Yunnan	Japonica rice
137	LR-145	Jiegunuo	Yunnan	Japonica rice
138	LR-146	LR16	Yunnan	Indica rice
139	LR-147	Long 16-3	Heilongjiang	Japonica rice
140	LR-148	Zhenzhu	Heilongjiang	Japonica rice
141	LR-149	Long 18	Yunnan	Japonica rice
142	LR-150	LR-139	Yunnan	Indica rice
143	LR-151	LR-140	Yunnan	Japonica rice
144	LR-152	LR-141	Yunnan	Japonica rice
145	LR-153	LR-142	Yunnan	Indica rice

### Appendix A.2

**Table A2:** Phenotypic data of germplasm.

Serial Number	TGW (g)	GL (mm)	GW (mm)	GS	GT (mm)	GD (g/L)	BRY (%)	MRR (%)	HRY (%)	GLMR (mm)	GWMR (mm)	MRGS	GTMR (mm)	TGWMR (g)	GDMR g/L
LR-1	21.74	7.67	3.44	2.23	2.01	410.97	75.21%	65.12%	60.28%	5.49	2.78	1.97	1.75	17.12	638.65
LR-2	19.68	7.02	3.14	2.24	1.94	460.02	73.01%	63.58%	61.88%	4.83	2.72	1.77	1.67	15.77	716.54
LR-3	23.58	6.86	3.45	1.99	2.18	457.50	77.58%	67.91%	64.20%	5.09	2.78	1.83	1.79	19.58	772.57
LR-4	19.55	6.77	3.49	1.94	2.06	402.37	70.62%	61.50%	60.39%	4.99	2.84	1.75	1.72	16.55	679.25
LR-6	28.07	8.03	3.88	2.07	2.22	405.10	77.74%	66.87%	65.18%	5.88	2.97	1.98	1.89	21.28	644.84
LR-7	26.96	7.68	3.46	2.22	2.18	466.09	77.24%	65.71%	61.85%	5.43	2.66	2.04	1.82	19.93	761.61
LR-8	23.49	6.86	3.43	2.00	2.16	462.54	78.74%	68.03%	64.42%	5.08	2.71	1.87	1.79	18.65	756.29
LR-9	24.09	6.83	3.44	1.98	2.09	489.24	80.22%	69.56%	66.80%	4.98	2.78	1.79	1.79	19.07	770.04
LR-10	22.75	7.67	3.44	2.23	1.97	438.85	74.08%	64.77%	60.60%	5.63	2.58	2.18	1.69	17.33	704.42
LR-11	18.56	6.27	3.62	1.73	2.00	408.64	73.25%	64.15%	62.72%	4.73	2.89	1.64	1.76	15.97	663.62
LR-12	24.68	7.28	3.65	1.99	2.14	433.82	74.28%	64.60%	62.41%	5.26	2.86	1.84	1.85	20.37	730.83
LR-13	25.26	8.84	3.31	2.67	1.96	440.68	76.31%	67.35%	65.75%	6.22	2.48	2.51	1.59	17.83	725.99
LR-14	24.98	8.32	4.05	2.05	2.41	307.42	74.59%	57.96%	52.45%	5.42	2.96	1.83	2.11	21.52	633.23
LR-15	26.81	7.94	3.97	2.00	2.24	379.57	73.97%	59.53%	51.68%	5.08	2.76	1.84	2.00	20.07	718.14
LR-16	24.55	7.77	3.45	2.25	1.96	466.17	78.75%	67.07%	65.14%	5.49	2.69	2.04	1.71	17.47	691.53
LR-17	19.62	6.59	3.44	1.91	2.17	398.01	79.96%	67.76%	66.41%	4.42	2.85	1.55	1.81	14.80	646.98
LR-18	19.35	6.72	3.61	1.86	2.13	374.54	78.59%	67.81%	66.45%	4.37	2.78	1.57	1.84	15.93	714.64
LR-19	24.63	8.33	3.39	2.46	2.03	430.81	74.01%	60.95%	51.82%	5.58	2.70	2.07	1.71	18.52	716.60
LR-20	24.37	8.71	3.04	2.86	2.00	458.85	77.72%	67.55%	64.78%	6.47	2.22	2.91	1.68	18.55	766.65
LR-22	24.06	7.37	3.42	2.16	2.07	460.59	77.89%	69.11%	66.53%	5.41	2.83	1.91	1.87	18.05	632.32
LR-23	24.16	7.51	3.47	2.17	2.02	459.53	77.48%	67.66%	64.57%	5.41	2.73	1.98	1.78	17.73	672.05
LR-25	18.43	7.40	3.03	2.44	1.87	440.06	72.40%	66.03%	64.49%	5.35	2.38	2.25	1.56	14.55	736.12
LR-26	26.32	7.70	3.57	2.16	2.09	458.59	79.74%	68.52%	66.08%	5.60	2.94	1.90	1.72	20.22	712.22
LR-27	24.60	6.77	3.50	1.94	2.19	475.02	80.85%	67.64%	63.60%	5.26	2.98	1.76	1.86	18.73	643.90
LR-28	23.74	7.01	3.49	2.01	2.15	451.40	79.92%	65.40%	60.45%	5.03	2.76	1.82	1.84	18.70	731.83
LR-29	25.17	8.14	3.41	2.39	1.97	459.75	72.73%	60.21%	49.39%	5.46	2.78	1.96	1.73	17.77	674.35
LR-30	23.15	6.68	3.67	1.82	2.19	431.29	77.23%	65.19%	63.14%	4.98	2.88	1.73	1.77	17.95	707.87
LR-31	25.77	8.04	3.47	2.32	2.03	453.58	79.40%	66.33%	53.38%	5.62	2.79	2.01	1.72	20.33	752.89
LR-32	20.02	7.67	3.40	2.26	1.92	400.82	77.88%	68.15%	66.72%	5.47	2.64	2.07	1.62	15.65	669.41
LR-33	24.47	7.34	3.60	2.04	2.20	420.25	80.51%	64.53%	56.27%	5.15	2.84	1.81	1.79	17.53	668.49
LR-34	23.43	7.38	3.72	1.98	2.16	395.06	79.03%	65.04%	63.43%	4.87	3.03	1.61	1.81	17.30	650.38
LR-35	20.00	8.00	3.02	2.65	1.89	438.25	77.70%	63.78%	58.51%	5.52	2.60	2.13	1.48	14.05	662.89
LR-36	22.20	7.05	3.83	1.84	2.08	396.14	80.59%	60.68%	53.72%	4.71	2.96	1.59	1.73	15.07	624.93
LR-37	22.53	8.28	3.10	2.67	1.89	465.07	74.91%	65.95%	64.43%	5.76	2.46	2.35	1.65	17.45	745.52
LR-38	21.93	8.39	3.34	2.51	1.92	406.77	76.15%	67.21%	64.28%	5.72	2.46	2.32	1.58	16.92	760.13
LR-39	19.83	7.05	3.77	1.87	2.13	350.26	73.88%	66.39%	65.51%	4.86	3.08	1.58	1.96	17.02	579.18
LR-40	25.23	8.04	3.56	2.26	2.08	423.18	78.40%	66.44%	64.54%	5.46	2.73	2.00	1.74	18.35	708.65
LR-41	23.60	7.23	3.79	1.91	2.26	380.83	75.97%	64.83%	63.42%	4.94	3.18	1.55	1.80	18.48	655.66
LR-42	24.77	8.77	3.46	2.53	2.02	403.70	78.70%	71.61%	69.70%	6.11	2.80	2.18	1.76	20.23	671.41
LR-43	24.68	8.72	3.34	2.61	2.07	409.97	78.36%	71.96%	69.84%	5.98	2.63	2.28	1.74	20.33	741.15
LR-44	20.90	6.94	3.57	1.94	1.99	422.38	68.23%	59.62%	57.21%	4.82	2.77	1.74	1.84	15.87	645.20
LR-45	26.95	7.13	3.61	1.98	2.15	486.97	79.21%	68.09%	65.91%	4.78	2.86	1.67	1.91	17.45	667.30
LR-46	21.28	6.78	3.50	1.94	2.13	420.66	79.69%	71.79%	68.97%	4.83	2.88	1.68	1.86	17.78	687.00
LR-48	23.43	7.53	3.39	2.22	2.13	431.93	80.10%	66.01%	62.56%	5.30	2.77	1.92	1.76	17.25	668.24
LR-49	19.78	6.73	3.70	1.82	2.06	386.98	78.66%	67.00%	63.66%	4.64	2.93	1.59	1.80	15.95	652.15
LR-50	27.42	8.32	3.59	2.32	2.13	430.43	79.52%	69.51%	58.85%	5.91	2.72	2.17	1.72	20.85	754.06
LR-51	23.10	8.15	3.38	2.41	2.00	418.44	79.31%	64.47%	58.63%	5.47	2.59	2.12	1.69	16.50	691.75
LR-53	20.12	6.58	3.75	1.75	2.13	383.75	77.95%	69.52%	66.27%	4.59	2.99	1.54	2.12	18.72	642.78
LR-54	25.17	7.38	3.71	1.99	2.30	400.15	78.57%	71.05%	69.27%	5.34	2.79	1.92	2.02	19.98	666.59
LR-55	22.25	7.83	3.36	2.33	1.98	427.35	79.05%	65.60%	60.70%	5.53	2.67	2.07	1.70	16.85	672.86
LR-56	22.45	8.32	3.22	2.59	1.93	433.46	78.00%	66.63%	63.52%	5.69	2.46	2.31	1.59	16.08	721.40
LR-57	27.18	7.98	3.78	2.11	2.18	413.61	78.01%	65.42%	62.62%	5.72	3.01	1.90	1.85	19.62	615.25
LR-58	29.80	8.54	3.99	2.14	2.29	383.03	80.20%	73.12%	72.07%	5.82	3.19	1.83	2.02	24.33	648.45
LR-59	25.25	7.56	3.65	2.07	2.21	415.08	78.95%	70.80%	67.52%	5.56	2.93	1.90	1.88	22.37	728.57
LR-60	24.23	8.26	3.11	2.66	2.06	456.85	80.36%	62.97%	54.28%	5.72	2.66	2.15	1.73	17.88	680.46
LR-61	20.08	8.00	3.33	2.40	1.89	398.20	74.82%	63.64%	59.03%	5.44	2.38	2.28	1.61	14.22	679.87
LR-62	24.08	8.23	3.44	2.40	2.04	418.00	77.81%	65.66%	60.62%	5.58	2.67	2.09	1.65	17.43	712.01
LR-63	23.33	7.12	3.56	2.00	2.17	423.97	79.65%	70.24%	69.38%	5.05	2.88	1.75	1.88	17.95	656.76
LR-64	22.18	6.61	3.67	1.80	2.23	409.68	81.91%	70.38%	67.59%	4.57	2.91	1.57	1.84	17.73	727.14
LR-65	23.73	7.42	3.78	1.96	2.15	394.32	80.48%	71.86%	69.45%	5.23	2.86	1.83	1.94	18.27	629.63
LR-66	24.90	7.91	3.86	2.05	2.20	371.42	79.96%	69.37%	65.04%	5.07	2.93	1.73	1.89	19.78	703.04
LR-67	24.85	8.37	3.36	2.49	2.06	429.98	78.53%	67.13%	64.99%	5.83	2.58	2.26	1.66	18.43	740.37
LR-68	29.27	8.52	3.52	2.42	2.14	455.77	80.13%	69.40%	65.44%	5.99	2.95	2.03	1.76	20.70	665.73
LR-69	22.20	7.12	3.39	2.10	2.16	426.00	82.38%	69.39%	67.87%	5.15	2.64	1.96	1.75	17.32	730.34
LR-70	18.48	7.23	3.22	2.24	1.93	411.78	77.34%	69.21%	67.39%	4.59	2.47	1.85	1.68	13.60	712.64
LR-71	26.78	8.54	3.40	2.51	2.02	456.28	80.67%	68.14%	65.36%	5.93	2.77	2.14	1.73	19.55	687.24
LR-72	26.00	8.44	3.41	2.48	2.00	452.96	80.50%	73.02%	71.90%	5.95	2.81	2.11	1.75	19.90	681.60
LR-73	26.20	8.41	3.39	2.49	2.00	460.72	79.95%	73.05%	72.56%	6.12	2.76	2.22	1.75	20.20	685.15
LR-74	27.80	8.68	3.57	2.43	2.07	433.89	78.18%	65.59%	61.19%	6.13	2.84	2.16	1.64	20.90	732.76
LR-75	22.65	7.20	3.51	2.05	2.14	419.33	77.57%	65.00%	64.00%	5.17	2.84	1.82	1.80	17.45	657.80
LR-76	32.13	10.04	4.01	2.51	2.16	369.51	78.26%	62.21%	52.50%	6.26	2.84	2.20	1.98	23.32	661.68
LR-77	23.67	7.78	3.25	2.39	2.07	451.73	77.80%	67.63%	65.40%	5.51	2.71	2.03	1.77	17.03	645.54
LR-78	26.68	7.97	3.52	2.26	2.10	452.14	80.07%	68.66%	64.05%	5.72	2.78	2.05	1.75	18.23	656.15
LR-79	21.52	7.27	3.65	1.99	2.21	367.59	72.62%	59.36%	55.62%	4.90	2.68	1.83	1.81	17.37	732.30
LR-80	25.85	8.26	3.42	2.41	2.07	442.45	75.14%	66.04%	64.24%	5.78	2.68	2.16	1.63	19.02	751.87
LR-81	24.43	7.38	3.66	2.02	2.14	422.14	79.17%	56.49%	39.96%	5.00	2.79	1.79	1.80	17.32	688.94
LR-82	26.88	8.54	3.61	2.36	2.09	417.68	78.53%	66.54%	64.15%	6.06	2.93	2.07	1.66	20.22	685.28
LR-83	26.92	8.08	3.54	2.28	2.11	445.92	80.61%	69.23%	63.71%	5.68	2.81	2.02	1.76	14.10	500.91
LR-84	26.80	8.46	3.66	2.31	2.06	420.82	76.39%	64.83%	60.85%	5.98	2.65	2.26	1.65	19.82	759.37
LR-85	22.77	6.59	3.66	1.80	2.36	399.26	82.36%	69.98%	66.95%	4.28	2.84	1.51	1.97	17.73	739.95
LR-86	25.02	7.58	3.46	2.19	2.00	476.88	79.99%	67.48%	64.00%	5.55	2.77	2.01	1.75	18.73	696.62
LR-87	24.93	6.90	3.83	1.80	2.19	430.66	80.62%	65.89%	61.69%	4.71	3.19	1.48	1.88	18.97	670.48
LR-88	22.83	6.95	3.97	1.75	2.26	366.15	78.82%	66.72%	64.01%	4.83	2.91	1.66	1.94	18.15	663.67
LR-89	23.53	7.41	3.45	2.15	1.99	462.61	81.74%	71.32%	69.94%	5.38	2.82	1.91	1.73	17.82	679.97
LR-90	28.65	8.75	3.62	2.42	2.07	437.95	79.59%	66.46%	60.30%	5.96	3.01	1.98	1.70	21.03	688.17
LR-91	24.13	7.46	3.75	1.99	2.26	381.57	79.04%	64.75%	62.52%	4.76	2.85	1.67	2.02	17.55	639.54
LR-92	25.85	8.25	4.16	1.98	2.23	337.42	79.03%	65.98%	60.19%	5.98	3.03	1.98	2.05	22.43	603.49
LR-93	26.58	8.54	4.23	2.02	2.19	335.83	80.16%	66.53%	57.39%	5.93	3.07	1.93	1.99	23.83	660.27
LR-94	26.07	8.49	4.11	2.06	2.25	332.44	78.57%	66.13%	60.27%	5.87	3.22	1.82	1.95	23.90	649.94
LR-95	22.65	7.48	3.33	2.24	2.04	446.28	80.50%	69.86%	69.77%	5.11	2.75	1.86	1.70	16.48	690.66
LR-96	22.95	6.65	3.46	1.92	2.30	433.16	83.84%	71.30%	65.88%	4.78	2.89	1.65	1.97	18.97	699.00
LR-97	17.97	6.44	3.62	1.78	2.02	382.73	75.83%	65.87%	64.75%	4.57	2.76	1.65	1.81	14.87	650.14
LR-98	24.92	7.90	3.44	2.30	2.04	448.77	79.94%	67.82%	59.27%	5.61	2.82	1.99	1.76	17.82	641.10
LR-99	24.90	8.02	3.56	2.25	2.02	431.00	80.21%	69.74%	64.74%	5.69	2.79	2.04	1.72	18.32	672.48
LR-100	24.27	8.03	3.85	2.09	2.09	375.69	81.57%	70.35%	59.73%	5.49	2.95	1.86	1.71	13.28	479.84
LR-102	20.50	6.48	3.87	1.67	2.11	387.80	76.41%	64.84%	61.79%	4.47	2.98	1.50	1.77	15.23	647.76
LR-103	23.90	7.53	3.48	2.16	2.22	411.07	79.23%	68.89%	65.49%	5.07	2.70	1.88	1.81	18.63	753.08
LR-104	24.17	7.38	3.56	2.07	2.25	407.50	80.02%	68.51%	65.79%	5.06	2.89	1.75	1.92	18.50	658.46
LR-105	25.03	8.40	3.13	2.68	2.05	463.65	80.50%	68.45%	63.04%	5.62	2.51	2.24	1.74	18.85	770.39
LR-106	24.85	8.35	3.20	2.61	2.03	458.91	79.24%	66.00%	55.50%	5.74	2.51	2.29	1.79	18.57	720.21
LR-107	25.63	7.88	3.56	2.21	2.06	444.14	78.99%	67.12%	64.88%	5.38	2.61	2.06	1.68	18.53	784.59
LR-109	20.00	7.68	3.53	2.17	2.07	356.95	69.07%	60.32%	59.28%	5.55	2.64	2.10	1.64	17.37	724.54
LR-110	20.82	7.81	3.06	2.55	1.92	453.37	79.60%	67.97%	65.25%	5.57	2.55	2.18	1.63	15.25	659.23
LR-111	22.12	7.67	3.16	2.43	1.92	474.90	80.02%	68.83%	64.05%	5.35	2.61	2.05	1.64	16.10	704.94
LR-112	22.78	7.31	3.32	2.20	2.21	426.15	81.92%	70.54%	69.39%	5.12	2.55	2.01	1.81	17.75	753.39
LR-113	25.95	8.53	4.05	2.10	2.08	361.27	77.10%	62.49%	58.01%	5.80	3.01	1.93	1.75	20.68	677.55
LR-114	26.13	8.60	4.02	2.14	2.10	359.83	77.89%	62.35%	58.72%	5.78	3.14	1.84	1.94	20.30	577.51
LR-115	23.67	8.55	3.03	2.82	1.96	464.84	76.94%	66.62%	63.22%	5.73	2.45	2.34	1.58	16.40	736.95
LR-116	31.13	7.90	3.68	2.15	2.36	452.18	85.41%	72.05%	57.54%	5.62	2.93	1.92	2.09	25.20	731.49
LR-117	23.20	7.69	3.49	2.21	2.14	405.33	79.43%	66.35%	62.23%	5.02	2.74	1.83	1.82	19.00	758.39
LR-118	23.00	7.80	3.33	2.34	2.27	390.36	81.04%	66.56%	64.29%	4.95	2.74	1.81	1.89	17.40	678.56
LR-119	28.40	8.39	3.50	2.40	2.07	466.47	78.17%	70.71%	68.72%	6.12	2.85	2.15	1.88	20.95	637.82
LR-120	26.33	7.55	3.99	1.89	2.23	392.40	81.34%	69.62%	61.43%	5.17	3.12	1.66	1.79	20.33	705.42
LR-122	28.77	8.90	3.45	2.58	2.03	462.70	78.53%	70.06%	68.36%	6.05	2.77	2.19	1.76	21.00	712.26
LR-123	23.53	7.44	3.24	2.30	2.22	439.80	84.96%	72.40%	68.44%	5.50	2.62	2.10	1.88	19.72	728.01
LR-124	25.50	8.36	3.24	2.58	2.10	446.71	78.50%	66.51%	59.86%	6.02	2.63	2.29	1.65	20.07	768.02
LR-125	22.95	7.03	3.83	1.84	2.15	397.07	78.02%	68.54%	67.17%	5.34	2.85	1.88	1.73	17.58	669.56
LR-126	22.73	7.67	3.47	2.21	2.31	370.06	82.67%	73.95%	72.85%	5.58	2.60	2.14	1.94	19.40	687.86
LR-127	22.27	7.46	3.52	2.12	2.16	393.21	78.79%	66.08%	63.24%	5.25	2.61	2.01	1.88	18.40	714.27
LR-128	23.57	7.55	3.72	2.03	2.13	394.26	80.75%	72.55%	70.98%	5.44	2.85	1.91	1.89	20.10	685.69
LR-129	29.99	8.65	3.37	2.56	2.11	487.55	79.08%	69.92%	67.07%	6.43	2.74	2.34	1.83	23.80	738.10
LR-130	21.90	7.48	3.61	2.07	2.13	379.98	75.42%	63.96%	62.34%	5.20	2.81	1.85	1.79	17.78	680.69
LR-131	19.75	6.82	3.88	1.76	2.08	358.86	73.57%	58.77%	55.13%	4.72	2.90	1.63	1.85	17.37	687.01
LR-132	24.87	8.36	3.72	2.25	2.11	378.56	76.20%	69.38%	66.84%	5.80	2.93	1.98	1.74	20.12	680.22
LR-133	28.63	7.62	4.06	1.88	2.37	390.16	82.33%	70.78%	69.84%	4.99	3.23	1.55	1.94	21.87	699.18
LR-134	24.07	7.18	3.99	1.80	2.22	378.75	79.49%	66.44%	64.85%	4.98	3.04	1.64	1.92	18.38	633.27
LR-135	23.85	8.66	3.02	2.87	2.12	430.21	81.96%	74.79%	74.03%	6.14	2.35	2.61	1.86	20.20	751.37
LR-136	26.65	9.24	3.42	2.70	2.02	418.44	77.50%	66.25%	57.04%	6.64	2.52	2.63	1.73	20.83	717.30
LR-137	21.07	6.30	3.46	1.82	2.15	449.20	82.64%	73.01%	72.30%	4.36	2.76	1.58	1.92	16.38	708.50
LR-138	25.10	9.08	3.32	2.73	2.13	390.67	78.63%	70.28%	65.91%	6.44	2.47	2.61	1.91	22.05	726.93
LR-139	24.93	8.68	3.25	2.67	2.18	406.70	82.59%	76.54%	67.58%	6.20	2.43	2.55	1.98	20.27	680.46
LR-140	21.95	6.73	3.50	1.92	2.20	424.07	83.10%	76.78%	76.58%	4.47	2.80	1.60	1.93	17.62	730.77
LR-141	28.10	9.12	3.28	2.78	2.18	430.09	81.87%	73.00%	48.01%	6.34	2.42	2.62	1.85	21.07	742.63
LR-142	22.97	8.75	3.00	2.92	2.04	429.52	79.28%	71.78%	71.32%	5.89	2.37	2.49	1.78	17.75	717.21
LR-143	19.88	8.09	3.44	2.35	1.97	361.77	77.61%	64.51%	61.87%	5.42	2.47	2.19	1.66	14.38	647.53
LR-144	26.55	8.19	4.08	2.01	2.17	366.44	76.69%	61.32%	55.44%	5.50	3.07	1.79	1.83	21.23	685.55
LR-145	18.70	8.12	3.35	2.42	1.87	367.63	71.72%	60.98%	54.08%	5.38	2.34	2.30	1.53	13.43	695.49
LR-146	25.58	7.83	3.58	2.19	2.02	452.74	80.06%	69.13%	63.76%	5.64	2.69	2.10	1.77	18.77	700.03
LR-147	26.17	10.00	2.93	3.41	1.95	458.18	82.42%	75.63%	74.08%	7.42	2.30	3.22	1.74	20.95	703.88
LR-148	19.30	8.49	2.63	3.23	1.87	463.08	77.06%	67.85%	67.51%	6.22	2.02	3.08	1.56	14.15	722.32
LR-149	29.08	9.86	3.45	2.86	2.12	403.85	77.87%	66.19%	63.30%	6.92	2.41	2.87	1.64	21.52	787.38
LR-150	16.60	6.86	3.52	1.95	1.99	345.66	68.73%	51.13%	47.66%	4.37	2.65	1.65	1.64	11.95	628.72
LR-151	22.88	7.45	3.97	1.88	2.32	333.64	75.33%	57.51%	53.20%	4.80	2.81	1.71	1.74	17.52	748.05
LR-152	26.05	9.11	3.01	3.02	2.03	467.26	79.24%	69.10%	66.18%	6.53	2.27	2.88	1.74	19.37	753.41
LR-153	22.63	9.21	3.61	2.55	2.03	335.71	75.37%	63.23%	59.71%	6.46	2.41	2.68	1.61	17.62	702.32

Note: The value is the average of three repetitions.
